# Acknowledgement to Reviewers of *Polymers* in 2016

**DOI:** 10.3390/polym9010023

**Published:** 2017-01-10

**Authors:** Polymers Editorial Office

**Affiliations:** MDPI AG, St. Alban-Anlage 66, 4052 Basel, Switzerland; polymers@mdpi.com

The editors of *Polymers* would like to express their sincere gratitude to the following reviewers for assessing manuscripts in 2016. 

We greatly appreciate the contribution of expert reviewers, which is crucial to the journal’s editorial process. We aim to recognize reviewer contributions through several mechanisms, of which the annual publication of reviewer names is one. Reviewers receive a voucher entitling them to a discount on their next MDPI publication and can download a certificate of recognition directly from our submission system. Additionally, reviewers can sign up to the service Publons (https://publons.com) to receive recognition. Of course, in these initiatives we are careful not to compromise reviewer confidentiality. Many reviewers see their work as a voluntary and often unseen part of their role as researchers. We are grateful to the time reviewers donate to our journals and the contribution they make. 

If you are interested in becoming a reviewer for *Polymers*, see the link at the bottom of the webpage http://www.mdpi.com/reviewers.

The following reviewed for *Polymers* in 2016:
Abbes, BLee, Wen-YaAbel, Steven M.Lehocky, MarianAbella, Laura SánchezLeón, GerardoAbenojar, J.Leporatti, StefanoAbou Neel, Ensanya A.Leung, Siu N.Adachi, NaoyaLi, AnlongAdachi, TakujiLi, DawenAdhikari, Birendra B.Li, JingAgliari, ElenaLi, LeiAhmed, MaryaLi, MingqiangAifantis, E. C.Li, Min-HuiAimi, JunkoLi, ShulongAkiba, IsamuLi, SumingAkitsu, TakashiroLi, WentaoAkiyoshi, KazunariLi, YingAlander, JarmoLi, YingzhenAlcoutlabi, MatazLi, ZibiaoAlhwaige, Almahdi A.Lianos, PanagiotisAl-Mosawe, AlaaLiao, SusanAlongi, JennyLignola, Gian PieroAltstädt, VolkerLin, Ching HsuanAmeli, AmirLin, Gwo-GengAmsden, Brian G.Lin, HaijingAndreev, Oleg A.Lin, Hsin-YiAndrews, MarkLin, Hung-YinAndrieux, FabriceLin, XiaojieAnnapureddy, VenkateswarluLin, Yang-weiAnraku, MakotoLing, Maurice HtAppelhans, DietmarLing, ShengjieArai, NoriyoshiLinkous, Clovis A.Arbelaiz, AitorLionetto, FrancescaArchodoulaki, Vasiliki-MariaLiu, DanqingAriga, KatsuhikoLiu, FeiArtiaga Díaz, RamónLiu, Fwu-HsingAsatekin, AyseLiu, JinglinAtzberger, PaulLiu, WendyAzoug, AurélieLiu, YinBaghi, HadiLiu, Ying-LingBai, JinboLoessner, DanielaBanobre, ManuelLoh, Xian JunBao, ChunhuiLolla, DineshBarbetta, AndreaLopes, Maria AscensãoBarborini, MatteoLou, TiejiongBarron, Andrew R.Low, Bee TingBashur, ChristopherLuo, JiangshuiBasiak, EwelinaLuong, Dzung DinhBasso, Maria CeciliaLuscombe, ChristineBayer, BernhardLusi, MatteoBeall, Gary W.Luzi, FrancescaBear, JosephLvov, YuriBechelany, MikhaelMa, JeffBella, FedericoMa, XingBelloncle, ChristopheMaas, MichaelBen Messaoud, GhaziMaeda, IsamuBencardino, FrancescoMahnken, RolfBenetatos, PanayotisMaier, GerhardBenito, Juan M.Maki, JamesBenyoucef, AbdelghaniMalinauskas, MangirdasBéreaux, Y.Maloney, MarkBeris, AntonyMalucelli, GiulioBernards, MattMan, DulaBerret, Jean-françoisManera, M.G.Berron, BradMarcal, HelderBeverina, LucaMarcinko, Joseph J.Bhowmick, SankhaMarlow, FrankBianco, I.D.Marrocchi, AssuntaBijwe, JayashreeMartino, SabataBikiaris, Dimitrios N.Martins, AlbinoBitterwolf, Thomas E.Mascia, LenoBlanco, IgnazioMascotto, SimoneBogatko, StuartMatsuda, YasuhiroBohayra Mortazavi, BohayraMatsumoto, AkikazuBoluk, YamanMatsumoto, Ken’ichiroBorbas, K. EszterMatsumura, ShuichiBorges, JoãoMatsuo, TakashiBorowska, MartaMatyjaszewski, KrzysztofBosch, P.Mawhinney, ThomasBose, Ranjita K.Mazzio, Katherine A.Bottari, GiovanniMcCarthy, EdwardBoucher, David S.McGuinness, DavidBoutinguiza, MohamedMedronho, BrunoBranco, FernandoMen, YongjunBrantley, William A.Ménard-Moyon, CéciliaBreinbauer, RolfMenendez, RosaBrighenti, RobertoMenger, FredricBrischetto, SalvatoreMerino, SoniaBronstein, HugoMessina, FrancescoBrosseau, ChristianMi, Fwu-LongBrovelli, SergioMicheli, DavideBrown, Eric N.Michels, JulienBrzezinski, RyszardMija, AliceBuchet, ReneMilner, Scott T.Bugg, Timothy D.H.Miomandre, FabienBurillo, GuillerminaMiranda, Jose M.Burke, Anthony J.Miron, Richard J.Burkhardt, TheodoreMisra, R.D.K.Bychanok, DzmitryMiura, YoshikoCabanelas, Juan CarlosMiyako, EijiroCabedo, L.Monteil-Rivera, FannyCallahan, Laura SmithMoratti, SteveCallan, BridgeenMorgan, AlexanderCametti, C.Mori, WasukeCancelliere, PiergiacomoMorimura, ShigeruCapacchione, CarmineMorris, EdwinCaraglio, MicheleMoseke, ClausCarosio, FedericoMoshaverinia, AlirezaCarter, KimberlyMosquera, Maria JesusCarvelli, ValterMotta, AntonellaCasado-Coterillo, ClaraMuller, AlejandroCasalini, TommasoMüller, Axel H. E.Caschera, DanielaMüller, BertCasettari, LucaMüller, Kei W.Castano, Lina M.Müller-Buschbaum, PeterCatanzano, OvidioMuralidhar, AbhiramCavalli, RobertaMurugan, N. ArulCavicchi, KevinMusumeci, TeresaCerruti, PierfrancescoNagai, KeijiCesano, FedericoNagao, DaisukeChandrawati, RonaNagaraj, Vinay J.Chang, Young-WookNagasaka, MasanariChang, YungNair, MridulaChapartegui, M.Nakamura, YoshinobuChartoff, RichardNarayanan, GaneshChase, George G.Narita, TetsuharuChen, BanglinNetz, RolandChen, Bor-kuanNgai, ToChen, Chuh-YungNgan, A.H.W.Chen, Jhy-DerNganga, SaraChen, JuanNguyen, PhuongChen, JunNiidome, TakuroChen, Jyh-chienNima, ZeidChen, Jyh-PingNischang, IvoChen, NingNishikitani, YoshinoriChen, Shin-JuhNisticò, NicolaChen, Yeng-LongNisticò, RobertoCheng, Chih-ChiaNitschke, MirkoCheng, Huai NNoël, MartinCheng, XingguoNoguchi, HiroshiCheng, Yang-tseNomura, K.Cheong, Yuen-KiNoonan, KevinChiang, Chin-LungNorvez, SophieChiappone, AnnalisaNotta-Cuvier, DelphineChieruzzi, ManilaNouvel, CécileChiessi, EsterNuhn, LutzCho, Dong-WooNumata, KeijiChoi, Jong-ilNune, Satish K.Chrissopoulou, KiriakiNuxoll, EricChristen, ThomasOaki, YuyaChung, Chan-MoonObata, MakotoChung, Tai-ShungOcando, ConnieCiardelli, FrancescoOgawa, AkiyaCicogna, FrancescaOh, IlkwonCinacchi, GiorgioOh, Jae-MinCipelletti, LucaOh, Se HeangCirillo, GiuseppeOhara, KeishiCladera, AntoniOka, ChiemiClausell, CarolinaOkamoto, MasamiCobo, AlfonsoOliva, LeoneCoelho, MárioOmbres, LucianoCohn, DanielOmi, ShinzoCollins, MauriceOppelt, Kerstin T.Colombi, PierluigiOren, YoramContreras-Cáceres, RafaelOrtenzi, Marco A.Cools, PieterOsakada, KohtaroCorcione, Carola EspositoPacheco, João G.Cordier, StéphanePack, SeongchanCorriou, Jean-PierrePackirisamy, MuthukumaranCosta, RuiPalama, Ilaria E.Covas, JosèPalmieri, Frank L.Cox, SophiePan, GuoqingCrook, VincentPantoya, Michelle L.Crouzier, ThomasPao, Chun-WeiCui, HaitaoPaolucci, GinoCunningham, Michael F.Papaccio, GianpaoloDa Silva, MarceloPapadakis, ChristineDae, ByundPapageorgiou, George Z.Dai, LiangParikka, KirstiDarvishmanesh, SiavashParisi, CristianDavis, FredPark, Cheol-MinDe La Flor, SilviaPark, HyunDe With, G.Park, Jong-SeokDeguchi, TetsuoPark, SukheeDel Gaudio, CostantinoPassaglia, ElisaDel Valle, Luis J.Passalacqua, AlbertoDeng, YongmingPatenaude, MathewDevie, ArnaudPatra, DebabrataDhakshinamoorthy, AmarajothiPatterson, JenniferD'hooge, DagmarPauer, WernerDi Bella, GuidoPaul, WolfgangDi Ludovico, MarcoPavlidis, IoannisDi Maio, LucianoPayne, ChristineDias, JulianaPellis, AlessandroDias, Rolando CSPeng, Chi-HowDietrich, Philipp-ImmanuelPeng, ErwinDing, JianxunPeng, RobertDionisi, DavidePereira, Ricardo N.Dixon, David A.Peresin, SoledadDobrzynski, PiotrPerrin, LaraDodou, KalliopiPeterson, AmyDolgushev, MaximPethrick, Richard A.Doll, KennethPetkova, DianaDomenici, ValentinaPetukhov, Andrei V.Dominguez, Juan CarlosPfeifer, Christian G.Don, Trong-MingPiemonte, V.Dongre, PrajaktaPinho, EvaDonnelly, Ryan F.Pissis, PolycarposDorfs, DirkPistone, AlessandroDotan, AnaPitsikalis, MarinosDragoni, EugenioPitto-Barry, AnaïsDrummond, CarlosPizzo, BenedettoDu, GuanbenPK, DuttaDuong, Hai M.Plamper, FelixDupuis, RaphaëlPlante, Jean-SébastienDuvigneau, JoostPlummer, John ChristopheDworak, AndrzejPojman, John A.Dziubla, ThomasPokorny, MarekEbara, MitsuhiroPolson, JamesEguiazábal, José I.Pomposo, José A.Eiser, ErikaPons, JosefinaEl-Lateef, Hany M. AbdPopescu, CosminElling, LotharPortugal, CarlaEndruweit, AndreasPotirniche, GabrielErbaş, AykutPoulikakos, LilyErdem, EmrePouponneau, PierreEstelrich, JoanPresa Soto, AlejandroEwen, JamesPrescott, StuartFaccio, GretaPretsch, ThorstenFagan-Murphy, AidanPrice, William E.Faggio, CaterinaPrud'homme, Robert E.Fang, StevenPucci, AndreaFare, SilviaPuiggalí, J.Faria, H.Puiggalí, JordiFatyeyeva, KaterynaPulati, NuerxidaFellows, ChrisPunta, CarloFelner, IsraelPunzo, FrancescoFeng, QuanyouPuyvelde, Peter VanFeng, YingQi, GuangyanFernandes, Emanuel M.Qiao, KunFernandes, SuseteQin, YangFernandez-Garcia, MartaQuijada-Garrido, IsabelFerrara, LorenzoQuirino, RafaelFerretti, ValeriaRagaert, KimFerrier, E.Ragauskas, ArthurFigueira, RitaRahier, HubertFiore, VincenzoRajan, VarunFleury, GuillaumeRam, Manoj K.Florczyk, Stephen J.Ramanavicius, ArunasFollain, NadègeRamani, AlwarFörsth, MichaelRamezani Kakroodi, AdelFoti, DoraRamidi, PunnamchandarFraaije, MarcoRamos, IdaliaFrank, ErikRana, DipakFranssen, Maurice C.R.Rao, AbhijitFrazier, CharlesRasekh, ManoochehrFrigione, MariaenricaRaucci, MgFrihart, Charles R.Razal, JoselitoFrydrych, IwonaRegev, OrenFu, QiangReilly, GwendolenFukuda, TakeshiRemerie, KlaasFurukawa, ReiRennhofer, HaraldFurusawa, HiroyukiRescignano, NicolettaFustin, Charles-andréReyes-Labarta, J. A.Gaan, SabyasachiReynisson, JóhannesGadgil, BhushanReynolds, Melissa M.Ganachaud, FrancoisRibeiro, Maria C.S.Gándara, FelipeRiguera, RicardoGarbovskiy, YuriyRissanou, Anastassia N.Garcia Ivars, JorgeRitchie, ChristopherGarcia, IñakiRitter, HelmutGarcia-Gonzalez, CarlosRoberson, David A.Gary-Bobo, MagaliRochat, SébastienGates, ByronRodrigue, DenisGe, TingRodriguez, Hortensia M.Gebel, GerardRodríguez, Julio R.Gellerstedt, GöranRodríguez-Couto, SusanaGenchi, GiadaRodríguez-López, JuliánGentile, PiergiorgioRodriguez-Ropero, FranciscoGeorge, Thomas F.Roger, LeblancGhafoori, ElyasRonca, AlfredoGhandi, KhashayarRonca, SaraGhiringhelli, LucaRoth, BernhardGhoshal, SushantaRottler, JoergGhoufi, AzizRousakis, TheodorosGiacometti, AchilleRovigatti, LorenzoGigli, MatteoRubenstein, David A.Gimenez-Pinto, VianneyRubio, Ramon G.Giordano, StefanoRusso, PietroGiraud, StephaneRyu, Sung HunGirousi, StellaSacco, AdrianoGizdavic-Nikolaidis, Marija R.Sada, KazukiGloria, AntonioSaito, ReikoGoh, Kheng LimSalaün, FabienGoh, Suat HongSalerno, AurelioGoñi, AsierSalerno, MarcoGonzalez-Diaz, HumbertoSameoto, DanGonzález-Fonteboa, BelénSamios, JannisGooneie, AliSamperi, FilippoGorgutsa, StepanSanli, AbdulkadirGorman, Christopher B.Santoni, Brandon G.Gorrasi, GiulianaSantoni, IlariaGossage, R. A.Santulli, CarloGoto, AtsushiSaraiva, M. Lúcia M.f.s.Gotor-Fernández, VicenteSarasini, FabrizioGouveia, SusanaSarasua, Jose-RamonGrassi, GabrieleSarazin, YannGraziani, AndreaSardon, HaritzGrenha, AnaSasan, NouranianGrisi, FabiaSavi, PatriziaGroot, RobScardamaglia, MattiaGu, YibeiSchartel, BernhardGuelcher, Scott A.Schauer, CarolineGui, MinghuiScheffer, TobiasGun’ko, YuriiSchirhagl, RomanaGuo, HuiminSchirp, ArneGupta, SanjuSchlaad, HelmutGutierrez, JunkalSchmid, MarkusGuzmán, EduardoSchröder, HendrikHaaparanta, Anne-MarieSchroeder, CharlesHager, MartinSchwarz, SimonaHamada, HiroyukiSchwendicke, FalkHammer, BrentonSebastien, BernacchiHampp, NorbertSeidel, Rüdiger W.Han, Woo-SuckSena-Cruz, JoseHao, XiaoqiongSenyuk, BohdanHaque, AnwarulSeo, Soo-YeonHarding, IanSerizawa, TakeshiHardwick, LaurenceSerra, AngelsHardy, JohnShahverdi, M.Harmandaris, VagelisShanbhag, SachinHarrisson, SimonShanmugharaj, A. M.Hasan, TawfiqueShapter, Joe G.Hashidzume, AkihitoShau, Min DaHashizume, MineoSheiko, Sergei S.Haspel, NuritShen, ChangshengHassan, MohammadShen, Kelvin K.Hayakawa, TohruShen, Yung-KangHayashi, ShotaroShendruk, TylerHe, GuoweiShenhar, RoyHe, QingliangSherborne, ColinHe, ZhongqiShih, Wen-PinHead, David A.Shimoi, NorihiroHeise, AndreasShinzawa, HideyukiHellweg, ThomasShopova, Siyka IHennig, AndreasShoseyov, OdedHess, SiegfriedShrestha, Lok KumarHideto, MinamiShrivastava, AbhishekHilber, WolfgangSikorska, HannaHildner, RichardSilva, Karnika DeHirayama, FumitoshiSingh, GurpreetHisada, KenjiSkov, AnneHo, Ko-ShanSliozberg, Yelena R.Hodge, PhilipSloop, JosephHofmann, UteSmith, Adam E.Hollen, Shawna M.Smith, AlanHong, LiangSobarzo-Sánchez, EduardoHong, SeonkiSödergård, AndersHorrocks, A. RichardSohn, Youn SooHorrocks, RichardSong, ChangsikHorsch, MartinSong, Jung-IlHorsky, JiřiSong, KenanHossain, M. E.Sonnier, RodolpheHou, ChongSorarù, Gian DomenicoHousecroft, Catherine E.Sotta, PaulHowell, Bob A.Sousa, AngelaHowlin, BrendanSPINA, RobertoHozumi, AtsushiStamatatos, Theocharis C.Hsiao, Pai-YiStapf, SiegfriedHsiao, Sheng-HueiSteinfeldt, NorbertHsu, Hsiu-FuStevanovic, TatjanaHu, JinlianStoychev, GeorgiHuang, Chien-LinStoykovich, Mark P.Huang, Chih-FengStrandman, SatuHuang, Chun-JenStrano, MichaelHuang, JianweiStrube, Oliver I.Huda, Sumaiya BinteSu, Hai-ChingHung, AndrewSu, JingHwang, Nathaniel S.Su, Wen-TaHwang, Do-HoonSu, ZhouchengHwang, Ye-JinSubramanian, VivekanandanIatridi, ZacharoulaSuckeveriene, RanIatrou, HermisSuckling, IanIglesias, MartaSugimoto, YoshikiIlg, PatrickSugiyasu, KazunoriIlles, ErzsebetSukhishvili, SvetlanaImbrogno, AlessandraSuryanto, BennyInomata, KatsuhiroSuzuki, DaisukeIraqi, AhmedSwindle-Reilly, Katelyn E.Irle, MarkSzekely, GyorgyIrusta, LourdesTabata, YasuhikoIshikawa, DataroTada, NaoyaISHIKAWA, ToshiyukiTAKADA, TomoyaItkis, Mikhail E.Takahashi, FumiakiIwata, TadahisaTakashima, KazutoJabbarzadeh, AhmadTakasu, AkinoriJacobsen, Jesper LykkeTakeshita, HirokiJain, AtulTan, Amy G.Y.Jajam, Kailash C.Tang, JieJäkle, FriederTanks, JonathonJan, Jeng-shiungTaratula, OlenaJaniak, ChristophTathireddy, PrashantJarvis, KarynTauer, KlausJavadi, AliTenhu, HeikkiJayant, Rahul DevTeramoto, NaozumiJayasinghe, SuwanTerao, KenJelinek, RazTextor, TorstenJenn-jong, YoungThakur, Vijay KumarJeong, ByeongmoonThickett, Stuart C.Jeong, Han MoThomas, SabuJessop, Julie L.p.Tiainen, HannaJiang, XianTilton, Robert D.Jiménez-Jorquera, CeciliaTobita, HidetakaJomaa, Mohamed HediToca-Herrera, José LuisJordi Aguilera-Sigalat, JordiTokita, MasatoshiJung, JaehanTomalia, DonaldJung, SeunhoTomlinson, DouglasJung, Woo JinTondi, GianlucaJunkers, ThomasTorres Lagares, DanielKadokawa, Jun-ichiTosello, GuidoKakkar, Ashok K.Tozzini, ValentinaKalantar Zadeh, KouroshTremblay, Marie-LaurenceKalogeras, Ioannis M.Trif, MonicaKamal Mohamed, Seeni MeeraTruong Phuoc, NghiaKan, Chi-waiTrzeciak, AnnaKanakubo, ToshiyukiTsakalof, AKanbara, TakakiTsang, Sai-WingKandare, EversonTsarkova, Larisa A.Kaneti, YusufTsitsilianis, ConstantinosKang, Jae-YoonTsui, C.P.Kang, Jeung-KuTsujimoto, TakashiKang, Sang WookTsurkan, Mikhail V.Karabiyik, UfukUnderwood, ShaneKarger-Kocsis, JózsefUnterreiner, Andreas-NeilKarpukhina, NataliaUr, SCKasal, BohumilVan Breemen, Lambert C.A.Katayama, YoshikiVan Griensven, MartijnKatz, Jonathan I.Van Herk, AlexKavalenka, Maryna N.Van Herwijnen, Hendrikus W. G.Kawaguchi, HarumaVan Steijn, VolkertKawahara, YutakaVancaeyzeele, CédricKawai, ToshiyukiVanderzande, Dirk J. M.Kawakatsu, ToshihiroVautard, FredericKawamura, IzuruVega, Juan F.Kenyon, William J.Venkataraman, ShrinivasKern, WolfgangVenugopal, GunasekaranKeul, HelmutVerbeek, JohanKhan, AnzarVirkki, JohannaKharlampieva, EugeniaVirnau, PeterKhokhlov, AlexeiVlassopoulos, DimitrisKhun, Nay WinVolkov, V.V.Kidoaki, SatoruVouyiouka, Stamatina N.Kihara, NobuhiroWadajkar, Aniket SKijima, MasashiWang, De-YiKim, Cheol SangWang, LiangKim, Hak-YongWang, Meng-jiyKim, Han-seungWang, QinyiKim, HeesunWang, ShuangyinKim, Jung BaeWang, TuoKim, SihwanWang, Tzu-WeiKim, Soo HyunWang, WenxinKim, SunjooWang, XiaKim, YoongWang, XuKim, Yoong AhmWare, TaylorKim, Young JunWarszynski, PiotrKimura, ShunsakuWatts, PeterKimura, YoshiharuWei, Kung-hwaKing, John T.Wei, QiangKiryukhin, Maxim V.Wei, YangKissin, Yury V.Wendel, Hans PeterKitazawa, TakafumiWeng, LihuiKiziltas, Esra ErbasWentzel, AlexanderKlaehn, John R.Wiesbrock, FrankKlapper, MarkusWildemann, BrittKlausen, R. S.Wilén, Carl-EricKlein, RolandWillerth, Stephanie MKo, Bao-TsanWittaker, AndrewKohlstedt, KevinWojkiewicz, Jean-LucKohri, MichinariWong, Shing-ChungKoizumi, ToshioWoo, Eamor MKojima, ChieWoodward, R. I.Kolbe, JanaWu, Shih-HsiungKoller, MartinWu, Tzi-YiKong, L.B.Wu, XiangfaKonnerth, JohannesWujcik, EvanKontou, E.Xavier, JoséKoper, Ger J. M.Xi, WeixianKornev, Konstantin (Kostya)Xia, YanKöster, SarahXiao, HaihuaKothapalli, Chandrasekhar R.Xiao, PuKotobuki, MasashiXu, Jun-TingKruk, MichalXue, LongjianKuang, HuihuiYachin, CohenKuckling, DirkYan, LiangKucuk, IsrafilYan, NingKuipers, Ernst J.Yan, XuehaiKukovec, Boris-MarkoYang, Po-ChihKulozik, UlrichYang, YongKuo, Dong-HauYang, ZhijunKuo, ShiaoweiYasuyuki, NakamuraKuršelis, KęstutisYeh, Jui-MingKwon, Il-KeunYeh, Yin-TingKwon, Tae-yubYelle, Daniel J.La Mantia, Francesco PaoloYoo, Byung-KukLadero Galán, MiguelYoon, Do Y.Lagowski, Jolanta B.Yoon, JinhwanLai, Jui-yangYoon, Moon-YoungLaiarinandrasana, L.Yoon, Soon-JongLamberti, LucianoYoshie, NaokoLamberti, MarinaYousif, BelalLang, GregorYoussef, Ahmed A.Lange, HeikoYu, JianLantada, Andres DiazYu, XinjunLarraneta Landa, EnekoYu, Yuan-HsiangLarson, RonaldYuen, Richard Kwok KitLau, Kim King-tongYusa, Shin-IchiLaurenzi, SusannaZacharia, NicoleLavilla, CristinaZajickova, ZuzanaLavina, SandraZaoutsos, StephanosLawler, JennyZarzar, Lauren D.Laycock, BronwynZazo, J.A.Lazzara, GiuseppeZelisko, Paul M.Lean, Meng H.Zhang, BoyuLeclère, PhilippeZhang, ChengfeiLecomte, PhilippeZhang, JianpingLee, Bor-ShiunnZhang, LongheLee, Bruce P.Zhang, QichunLee, Bun YeoulZhang, SanliangLee, Chang-SeopZhang, WenruiLee, Chia-hungZhao, BoxinLee, Hyung IlZheng, MinghaoLee, Jae-SukZhong, TuhuaLee, JaewoongZhou, HongLee, Jin WooZhou, JiajingLee, Jin-KyunZhou, RuhaiLee, JonghwiZhou, ZhengpingLee, JungwooZhu, XiangweiLee, SeungaeZille, AndreaLee, TaeyoonZinn, ManfredLee, Wen-Jau



